# Duloxetine ameliorates chronic stress-induced depressive behaviors by normalizing hippocampal SIK2-CRTC1 signaling

**DOI:** 10.3389/fphar.2026.1836082

**Published:** 2026-07-02

**Authors:** Hui Xu, Hua Fan, Bo Jiang, E-hui Ding, Peng Hou, Wei-bing Zhu

**Affiliations:** 1 Department of Neurosurgery, Nantong Hospital to Nanjing University of Chinese Medicine, Nantong, Jiangsu, China; 2 The First Affiliated Hospital, College of Clinical Medicine of Henan University of Science and Technology, Luoyang, Henan, China; 3 Department of Pharmacology, School of Pharmacy, Nantong University, Nantong, Jiangsu, China

**Keywords:** cAMP response element binding protein (CREB), CREB-regulated transcription co-activator 1, depression, duloxetine, hippocampus, salt-inducible kinase 2

## Abstract

**Introduction:**

Duloxetine, a serotonin-norepinephrine reuptake inhibitor, is clinically effective for major depressive disorder, yet the intracellular mechanisms underlying its therapeutic actions remain incompletely understood. The hippocampal salt-inducible kinase 2 (SIK2)-CREB-regulated transcription coactivator 1 (CRTC1) signaling cascade has been implicated in chronic stress-induced neuroplasticity deficits, but whether duloxetine engages this pathway to produce antidepressant effects is unknown.

**Methods:**

Male C57BL/6J mice were subjected to chronic social defeat stress, chronic unpredictable mild stress, or chronic restraint stress, followed by duloxetine treatment. Behavioral assessments included the forced swim test, tail suspension test, sucrose preference test, and social interaction test. Hippocampal tissues were analyzed for SIK2 and CRTC1 expression (protein and mRNA), CRTC1 subcellular distribution, and CRTC1–CREB interaction using western blotting, qRT-PCR, and co-immunoprecipitation. Adeno-associated virus-mediated hippocampal CRTC1 knockdown was employed to assess pathway requirement.

**Results:**

Duloxetine treatment effectively reversed chronic stress-induced behavioral abnormalities across all three models. At the molecular level, duloxetine normalized stress-induced upregulation of hippocampal SIK2 and downregulation of CRTC1, restored nuclear CRTC1 translocation, and enhanced CRTC1–CREB binding. Notably, hippocampal CRTC1 knockdown completely abrogated duloxetine’s behavioral effects.

**Discussion:**

These findings identify the hippocampal SIK2–CRTC1–CREB axis as a key downstream mediator of duloxetine’s antidepressant efficacy, expanding our understanding of how serotonin-norepinephrine reuptake inhibitors modulate neuroplasticity and providing a potential molecular target for antidepressant action.

## 1 Introduction

Major depressive disorder remains a leading cause of disability worldwide, affecting approximately 280 million people across all age groups (Kim et al., 2023; Ortega et al., 2022; Pastis et al., 2023). While the introduction of selective serotonin reuptake inhibitors and serotonin-norepinephrine reuptake inhibitors represented a significant advance over earlier tricyclic antidepressants, the therapeutic limitations of these agents—including delayed onset, insufficient response rates, and residual symptoms—point to the need for deeper understanding of depression pathophysiology (Boku et al., 2018; Gonda et al., 2023; Perez-Caballero et al., 2019). Contemporary research has shifted focus from neurotransmitter deficiencies toward disruptions in intracellular signaling pathways governing neuroplasticity, synaptic function, and neuronal survival (Mudgal et al., 2022; Price and Duman, 2020; Tartt et al., 2022).

Among brain regions implicated in mood regulation, the hippocampus has received considerable attention due to its vulnerability to chronic stress and its capacity for activity-dependent plasticity (Dean and Keshavan, 2017). Chronic stress-induced hippocampal dysfunction is characterized by reduced brain-derived neurotrophic factor (BDNF) signaling, impaired neurogenesis, and dendritic atrophy—all of which contribute to depressive symptomatology (Dean and Keshavan, 2017; Sheline et al., 2019). The transcription factor cAMP response element-binding protein (CREB) serves as a central hub integrating diverse signals that regulate BDNF expression and other plasticity-related genes (Amidfar et al., 2020). CREB activity is modulated by a family of coactivators known as CRTCs (CREB-regulated transcription coactivators), which shuttle between cytoplasm and nucleus in response to cellular signals (Altarejos and Montminy, 2011; Yin et al., 2022; Yoon et al., 2021).

Salt-inducible kinases (SIKs), particularly the SIK2 isoform, have emerged as important regulators of CRTC1 function (Darling and Cohen, 2021; Sasaki et al., 2011). Under basal conditions, SIK2 phosphorylates CRTC1, retaining it in the cytoplasm and preventing CREB activation (Sasaki et al., 2011). Chronic stress exposure upregulates hippocampal SIK2 expression, leading to enhanced CRTC1 phosphorylation, cytoplasmic sequestration, and consequent suppression of CREB-dependent gene transcription (Cai et al., 2024; Jiang et al., 2019). Genetic or pharmacological inhibition of SIK2 promotes CRTC1 nuclear translocation, restores CREB-mediated transcription, and produces antidepressant-like effects in preclinical models, establishing the SIK2-CRTC1-CREB pathway as a promising therapeutic target (Huang et al., 2023; Jiang et al., 2019; Liu et al., 2020).

Duloxetine, a dual reuptake inhibitor of serotonin and norepinephrine, is widely prescribed for major depressive disorder, generalized anxiety disorder, and neuropathic pain conditions (Bellingham and Peng, 2010; Carter and McCormack, 2009; Dhillon, 2013). Beyond its immediate effects on monoamine availability, duloxetine has been shown to influence neurotrophic factor expression and synaptic plasticity in limbic brain regions (Engel et al., 2013; Maciukiewicz et al., 2015; Xu et al., 2016). However, whether these effects involve modulation of the SIK2-CRTC1-CREB cascade has not been investigated. The present study was designed to address this gap by examining duloxetine’s impact on hippocampal SIK2-CRTC1 signaling in three complementary chronic stress models and determining the functional requirement of CRTC1 for its behavioral efficacy.

## 2 Materials and methods

### 2.1 Ethical statement

All experimental procedures were approved by the Institutional Animal Care and Use Committee of Nantong Hospital to Nanjing University of Chinese Medicine, and conducted in compliance with the ARRIVE 2.0 guidelines (Sprague, 2020). Efforts were made throughout to minimize animal suffering.

### 2.2 Animals

Eight-week-old male C57BL/6J mice (22–24 g) and retired male CD1 breeders (>9 months, >40 g) were obtained from SLAC Laboratory Animal Co., Ltd (Shanghai, China). Mice were maintained under controlled environmental conditions (12 h light/dark cycle, lights on 07:00; temperature 22 °C ± 2 °C; humidity 50%–60%) with free access to food and water. Following 1 week acclimatization, C57BL/6J mice were randomly assigned to experimental groups based on body weight stratification. Sample sizes were determined according to not only power analysis (α = 0.05, power = 0.80) but also many previous studies (Hua et al., 2021; Huang et al., 2020; Jiang et al., 2022; Shi et al., 2022; Su et al., 2019; Wu et al., 2020; Zeng et al., 2020; Zhang J. et al., 2020; Zhao J. et al., 2020; Zhao X. et al., 2020). Behavioral testing occurred during the light phase (09:00-17:00) by experimenters blinded to treatment conditions. For molecular analyses, mice were euthanized between 09:00-10:00 by decapitation under isoflurane anesthesia.

This study was conducted exclusively in male mice based on the following considerations. First, the three chronic stress paradigms employed have been extensively characterized in male C57BL/6J mice worldwide. Second, using male mice eliminates the confounding influence of estrous cycle-related fluctuations in ovarian hormones on both stress vulnerability and antidepressant responses, allowing for a more direct assessment of the SIK2-CRTC1 signaling pathway. Third, this initial mechanistic investigation was designed to establish proof-of-principle that duloxetine engages the hippocampal SIK2-CRTC1-CREB axis, which we viewed as a necessary prerequisite prior to extending the findings to females. Nevertheless, we fully acknowledge that the absence of female subjects represents a significant limitation of the present study (see Discussion).

### 2.3 Drug administration

Duloxetine hydrochloride (TargetMol, Boston, MA, USA) was dissolved in vehicle (5% dextrose in 0.9% saline containing 1% DMSO and 4% Cremophor EL, pH 7.0) and administered intraperitoneally at 10 mL/kg body weight. Doses (10 and 20 mg/kg) were selected based on published literature (Kato et al., 2022; Meejuru et al., 2021; Wang et al., 2025). Control mice received vehicle alone.

### 2.4 Chronic social defeat stress (CSDS)

The CSDS protocol was adapted from established methods (Hernandez Silva et al., 2024; Wang M. et al., 2021; Wang W. et al., 2021). Briefly, experimental C57BL/6J mice were introduced into the home cage of an unfamiliar and aggressive CD1 resident mouse for 10 min of direct physical interaction. Following the defeat session, the animals were separated by a perforated plexiglass divider, allowing continuous sensory contact for the remainder of the 24-h period. This procedure was repeated for 10 consecutive days, with a different CD1 aggressor each day. To ensure consistency, only CD1 mice that displayed persistent attack latencies of less than 60 s in three consecutive screening sessions were used. Non-stressed control mice were pair-housed with a same-sex conspecifics and handled daily. Drug treatments (duloxetine or vehicle) were administered once daily (between 8:00-9:00 a.m.) for another 2 weeks after CSDS exposure, and behavioral tests were conducted 24 h after the final injection.

### 2.5 Additional materials and methods

See the Supplementary Material for description of chronic unpredictable mild stress (CUMS), chronic restraint stress (CRS), forced swim test (FST), tail suspension test (TST), sucrose preference test (SPT), social interaction test, open field test (OFT), western blotting, quantitative real-time reverse transcription PCR (qRT-PCR), co-immunoprecipitation (Co-IP), and adeno-associated virus (AAV)-mediated gene transfer of short hairpin RNA (shRNA).

### 2.6 Statistical analysis

Data are expressed as mean ± S.E.M. Two-way ANOVA with Bonferroni’s *post hoc* test was used for comparisons involving two factors. One-way ANOVA with Tukey’s test was used where appropriate. Significance was set at *p* < 0.05.

## 3 Results

### 3.1 Duloxetine attenuates depressive-like behaviors in three chronic stress models

**FIGURE 1:**
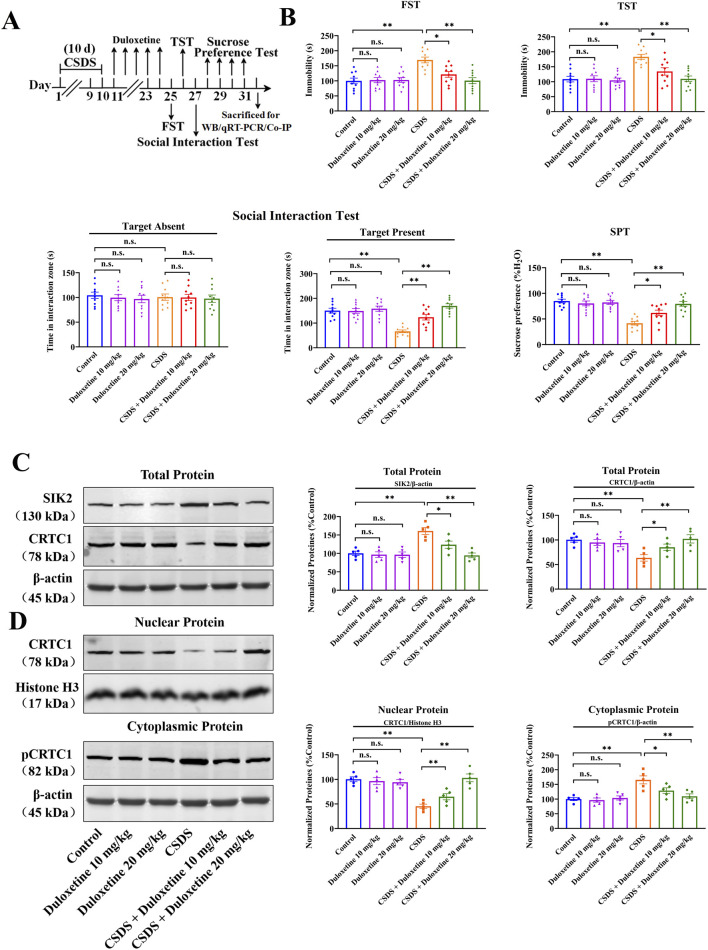
Duloxetine administration reverses CSDS-induced behavioral deficits and hippocampal SIK2-CRTC1 dysregulation. **(A)** Experimental timeline. **(B)** Duloxetine (10 and 20 mg/kg) treatment reduced FST and TST immobility, restored sucrose preference, and increased social interaction in CSDS-exposed mice (n = 10). **(C)** Representative blots and quantification showing that duloxetine treatment normalized CSDS-induced SIK2 upregulation and CRTC1 downregulation in the hippocampus (n = 5). **(D)** Subcellular fractionation revealed that duloxetine administration restored nuclear CRTC1 and reduced cytoplasmic pCRTC1 in the hippocampus of CSDS-exposed mice (n = 5). Data are presented as mean ± S.E.M.; *p < 0.05, **p < 0.01, n. s., not significant. Two-way ANOVA and Bonferroni’s test were adopted together.

**FIGURE 2:**
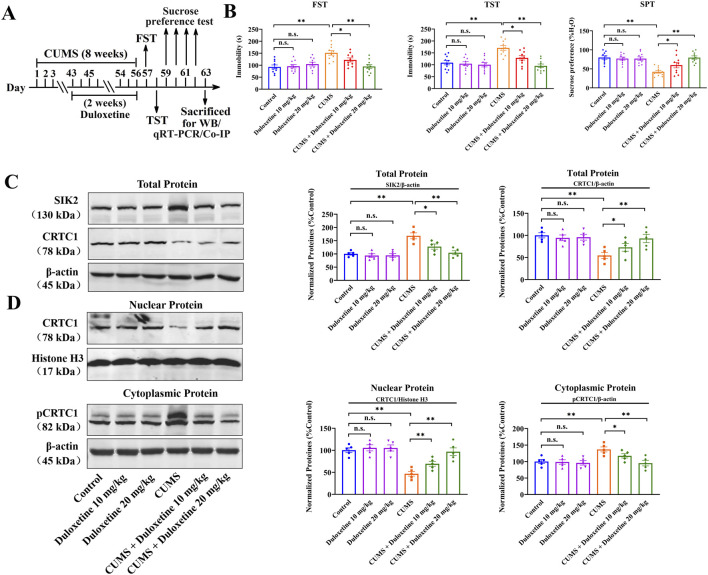
Duloxetine treatment attenuates CUMS-induced behavioral and molecular alterations. **(A)** Experimental timeline. **(B)** Duloxetine administration decreased FST/TST immobility and increased sucrose preference in CUMS-exposed mice (n = 10). **(C)** Duloxetine administration normalized CUMS-induced SIK2 upregulation and CRTC1 downregulation in the hippocampus (n = 5). **(D)** Duloxetine administration restored nuclear CRTC1 and reduced cytoplasmic pCRTC1 in the hippocampus of CUMS-exposed mice (n = 5). Data are presented as mean ± S.E.M.; *p < 0.05, **p < 0.01, n. s., not significant. Two-way ANOVA and Bonferroni’s test were adopted together.

**FIGURE 3:**
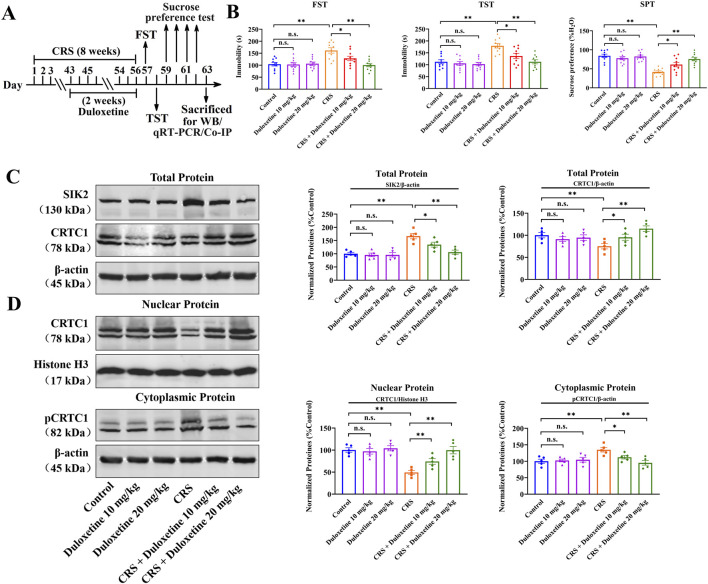
Duloxetine injection ameliorates CRS-induced depressive-like behaviors and molecular changes. **(A)** Experimental timeline. **(B)** Duloxetine injection reduced FST/TST immobility and increased sucrose preference in CRS-exposed mice (n = 10). **(C)** Duloxetine injection normalized CRS-induced SIK2 upregulation and CRTC1 downregulation in the hippocampus (n = 5). **(D)** Duloxetine injection restored nuclear CRTC1 and reduced cytoplasmic pCRTC1 in the hippocampus of CRS-exposed mice (n = 5). Data are presented as mean ± S.E.M.; *p < 0.05, **p < 0.01, n. s., not significant. Two-way ANOVA and Bonferroni’s test were adopted together.

We first evaluated whether duloxetine treatment ameliorates stress-induced behavioral deficits. In CSDS-exposed mice, 2 weeks of duloxetine administration (10 or 20 mg/kg) significantly reduced immobility time in both the FST [ANOVA: Stress F (1, 54) = 28.954, *p* < 0.01; Drug F (2, 54) = 23.601, *p* < 0.01; Interaction F (2, 54) = 17.838, *p* < 0.01] and TST [ANOVA: Stress F (1, 54) = 26.443, *p* < 0.01; Drug F (2, 54) = 21.392 *p* < 0.01; Interaction F (2, 54) = 16.282, *p* < 0.01], restored sucrose preference [ANOVA: Stress F (1, 54) = 30.204, *p* < 0.01; Drug F (2, 54) = 25.854, *p* < 0.01; Interaction F (2, 54) = 20.126, *p* < 0.01], and increased social interaction duration [ANOVA: Stress F (1, 54) = 46.557, *p* < 0.01; Drug F (2, 54) = 34.789, *p* < 0.01; Interaction F (2, 54) = 27.301, *p* < 0.01] compared to vehicle-treated stressed animals (n = 10, *p* < 0.01; Figures 1A,B). Similarly, in CUMS-exposed mice, duloxetine treatment decreased FST [ANOVA: Stress F (1, 54) = 26.849, *p* < 0.01; Drug F (2, 54) = 22.166, *p* < 0.01; Interaction F (2, 54) = 16.753, *p* < 0.01] and TST [ANOVA: Stress F (1, 54) = 31.587, *p* < 0.01; Drug F (2, 54) = 26.456, *p* < 0.01; Interaction F (2, 54) = 20.804, *p* < 0.01] immobility and enhanced sucrose preference [ANOVA: Stress F (1, 54) = 25.432, *p* < 0.01; Drug F (2, 54) = 22.144, *p* < 0.01; Interaction F (2, 54) = 18.257, *p* < 0.01] (n = 10, *p* < 0.01; Figures 2A,B). In CRS-exposed mice, duloxetine produced comparable behavioral improvements across all measures [ANOVA for FST: Stress F (1, 54) = 24.604, *p* < 0.01; Drug F (2, 54) = 20.368, *p* < 0.01; Interaction F (2, 54) = 14.971, *p* < 0.01. ANOVA for TST: Stress F (1, 54) = 29.436, *p* < 0.01; Drug F (2, 54) = 23.799, *p* < 0.01; Interaction F (2, 54) = 18.203, *p* < 0.01. ANOVA for SPT: Stress F (1, 54) = 27.349, *p* < 0.01; Drug F (2, 54) = 21.855, *p* < 0.01; Interaction F (2, 54) = 15.707, *p* < 0.01] (n = 10, *p* < 0.01; Figures 3A,B). The 20 mg/kg dose consistently showed robust efficacy, while duloxetine treatment had no effect on baseline behaviors in non-stressed controls (n = 10). We have also verified that general locomotor activity of mice among all groups was not affected, excluding the possibility that the observed changes in immobility in the FST and TST were due to motor impairment (n = 10; Supplementary Figure S1).

### 3.2 Duloxetine normalizes chronic stress-induced changes in hippocampal SIK2 and CRTC1 expression

Western blot analysis revealed that all three stress paradigms significantly upregulated hippocampal SIK2 protein levels and downregulated total CRTC1 protein levels (n = 5, *p* < 0.01; Figures 1C, 2C, 3C). Duloxetine treatment reversed these alterations, restoring both SIK2 and CRTC1 expression toward control levels (n = 5, *p* < 0.01; Figures 1C, 2C, 3C). For CSDS: SIK2 [ANOVA: Stress F (1, 24) = 35.467, *p* < 0.01; Drug F (2, 24) = 28.251, *p* < 0.01; Interaction F (2, 24) = 22.167, *p* < 0.01], total CRTC1 [ANOVA: Stress F (1, 24) = 27.376, *p* < 0.01; Drug F (2, 24) = 21.504, *p* < 0.01; Interaction F (2, 24) = 16.833, *p* < 0.01]. For CUMS: SIK2 [ANOVA: Stress F (1, 24) = 41.249, *p* < 0.01; Drug F (2,24) = 33.415, *p* < 0.01; Interaction F (2, 24) = 25.375, *p* < 0.01], total CRTC1 [ANOVA: Stress F (1, 24) = 29.869, *p* < 0.01; Drug F (2, 24) = 24.174, *p* < 0.01; Interaction F (2, 24) = 18.469, *p* < 0.01]. For CRS: SIK2 [ANOVA: Stress F (1, 24) = 35.745, *p* < 0.01; Drug F (2,24) = 30.432, *p* < 0.01; Interaction F (2, 24) = 22.386, *p* < 0.01], total CRTC1 [ANOVA: Stress F (1, 24) = 26.511, *p* < 0.01; Drug F (2, 24) = 21.309, *p* < 0.01; Interaction F (2, 24) = 16.245, *p* < 0.01].

**FIGURE 4:**
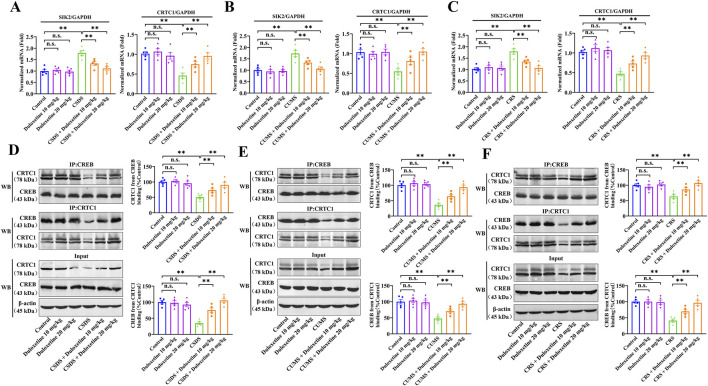
Duloxetine administration reverses chronic stress-induced transcriptional changes and restores hippocampal CRTC1-CREB interaction in stressed mice. **(A–C)** Results of qRT-PCR showing that duloxetine administration normalized the changes in hippocampal SIK2 mRNA (upregulation) and CRTC1 mRNA (downregulation) induced by CSDS **(A)**, CUMS **(B)**, and CRS **(C)** (n = 5). **(D–F)** Co-IP results demonstrating that duloxetine administration restored the decrease in CRTC1-CREB binding induced by CSDS **(D)**, CUMS **(E)**, and CRS **(F)** (n = 5). Data are presented as mean ± S.E.M.; **p < 0.01, n. s., not significant. Two-way ANOVA and Bonferroni’s test were adopted together.

Analysis of qRT-PCR demonstrated corresponding changes at the transcriptional level: stress exposure increased SIK2 mRNA and decreased CRTC1 mRNA, effects that were normalized by duloxetine treatment (n = 5, *p* < 0.01; Figures 4A–C). For CSDS: SIK2 mRNA [ANOVA: Stress F (1, 24) = 38.236, *p* < 0.01; Drug F (2, 24) = 30.425, *p* < 0.01; Interaction F (2, 24) = 24.103, *p* < 0.01], CRTC1 mRNA [ANOVA: Stress F (1, 24) = 29.785, *p* < 0.01; Drug F (2, 24) = 23.499, *p* < 0.01; Interaction F (2, 24) = 18.538, *p* < 0.01]. For CUMS: SIK2 mRNA [ANOVA: Stress F (1, 24) = 43.867, *p* < 0.01; Drug F (2,24) = 35.205, *p* < 0.01; Interaction F (2, 24) = 28.423, *p* < 0.01], CRTC1 mRNA [ANOVA: Stress F (1, 24) = 32.101, *p* < 0.01; Drug F (2, 24) = 26.345, *p* < 0.01; Interaction F (2, 24) = 20.796, *p* < 0.01]. For CRS: SIK2 mRNA [ANOVA: Stress F (1, 24) = 39.137, *p* < 0.01; Drug F (2,24) = 32.118, *p* < 0.01; Interaction F (2, 24) = 24.667, *p* < 0.01], CRTC1 mRNA [ANOVA: Stress F (1, 24) = 29.709, *p* < 0.01; Drug F (2, 24) = 24.019, *p* < 0.01; Interaction F (2, 24) = 17.178, *p* < 0.01].

Duloxetine did not alter these parameters in non-stressed mice (n = 5).

### 3.3 Duloxetine restores CRTC1 nuclear translocation and CRTC1-CREB interaction in the hippocampus of stressed mice

Subcellular fractionation showed that chronic stress significantly decreased nuclear CRTC1 while increasing cytoplasmic phosphorylated CRTC1 (pCRTC1) levels (n = 5, *p* < 0.01; Figures 1D, 2D, 3D). Duloxetine treatment fully reversed these changes, promoting CRTC1 nuclear translocation (n = 5, *p* < 0.01; Figures 1D, 2D, 3D). For CSDS: nuclear CRTC1 [ANOVA: Stress F (1, 24) = 33.158, *p* < 0.01; Drug F (2, 24) = 26.794 *p* < 0.01; Interaction F (2, 24) = 20.156, *p* < 0.01]; cytoplasmic pCRTC1 [ANOVA: Stress F (1, 24) = 28.702, *p* < 0.01; Drug F (2, 24) = 23.099, *p* < 0.01; Interaction F (2, 24) = 18.431, *p* < 0.01]. For CUMS: nuclear CRTC1 [ANOVA: Stress F (1, 24) = 44.165, *p* < 0.01; Drug F (2, 24) = 35.604, *p* < 0.01; Interaction F (2, 24) = 27.284, *p* < 0.01]; cytoplasmic pCRTC1 [ANOVA: Stress F (1, 24) = 36.045, *p* < 0.01; Drug F (2, 24) = 30.313, *p* < 0.01; Interaction F (2, 24) = 23.129, *p* < 0.01]. For CRS: nuclear CRTC1 [ANOVA: Stress F (1, 24) = 40.633, *p* < 0.01; Drug F (2, 24) = 31.351, *p* < 0.01; Interaction F (2, 24) = 25.795, *p* < 0.01]; cytoplasmic pCRTC1 [ANOVA: Stress F (1, 24) = 33.137, *p* < 0.01; Drug F (2, 24) = 28.082, *p* < 0.01; Interaction F (2, 24) = 21.804, *p* < 0.01].

Co-IP assays revealed that chronic stress exposure markedly reduced CRTC1-CREB binding in hippocampal nuclear extracts (n = 5, *p* < 0.01; Figures 4D–F). Duloxetine treatment restored this interaction, with the 20 mg/kg dose showing complete normalization (n = 5, *p* < 0.01; Figures 4D–F). Reciprocal Co-IP experiments (immunoprecipitating CREB and blotting for CRTC1) yielded consistent results (n = 5; Figures 4D–F). For CSDS: CRTC1 from CREB binding [ANOVA: Stress F (1, 24) = 32.805, *p* < 0.01; Drug F (2, 24) = 25.049, *p* < 0.01; Interaction F (2, 24) = 19.382, *p* < 0.01]; CREB from CRTC1 binding [ANOVA: Stress F (1, 24) = 31.344, *p* < 0.01; Drug F (2, 24) = 23.835, *p* < 0.01; Interaction F (2, 24) = 17.267, *p* < 0.01]. For CUMS: CRTC1 from CREB binding [ANOVA: Stress F (1, 24) = 36.189, *p* < 0.01; Drug F (2, 24) = 24.964, *p* < 0.01; Interaction F (2, 24) = 18.985, *p* < 0.01]; CREB from CRTC1 binding [ANOVA: Stress F (1, 24) = 30.104, *p* < 0.01; Drug F (2, 24) = 22.793, *p* < 0.01; Interaction F (2, 24) = 16.384, *p* < 0.01]. For CRS: CRTC1 from CREB binding [ANOVA: Stress F (1, 24) = 29.235, *p* < 0.01; Drug F (2, 24) = 24.046, *p* < 0.01; Interaction F (2, 24) = 15.405, *p* < 0.01]; CREB from CRTC1 binding [ANOVA: Stress F (1, 24) = 27.138, *p* < 0.01; Drug F (2, 24) = 21.565, *p* < 0.01; Interaction F (2, 24) = 14.253, *p* < 0.01].

### 3.4 Hippocampal CRTC1 is necessary for Duloxetine’s antidepressant effects

**FIGURE 5:**
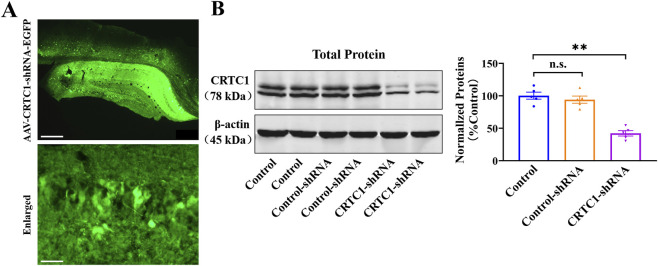
Validation of hippocampal CRTC1 knockdown by using AAV-CRTC1-shRNA-EGFP. **(A)** Representative fluorescence images showing AAV-mediated EGFP expression in the hippocampus of mice. Scale bars: 200 µm (Representative images) and 25 µm (Enlarged images). **(B)** Related western blotting results confirming that the usage of AAV-CRTC1-shRNA induced approximately 58% reduction in hippocampal CRTC1 protein expression (n = 5). Data are presented as mean ± S.E.M.; **p < 0.01, n. s., not significant. One-way ANOVA and Tukey’s test were adopted together.

**FIGURE 6:**
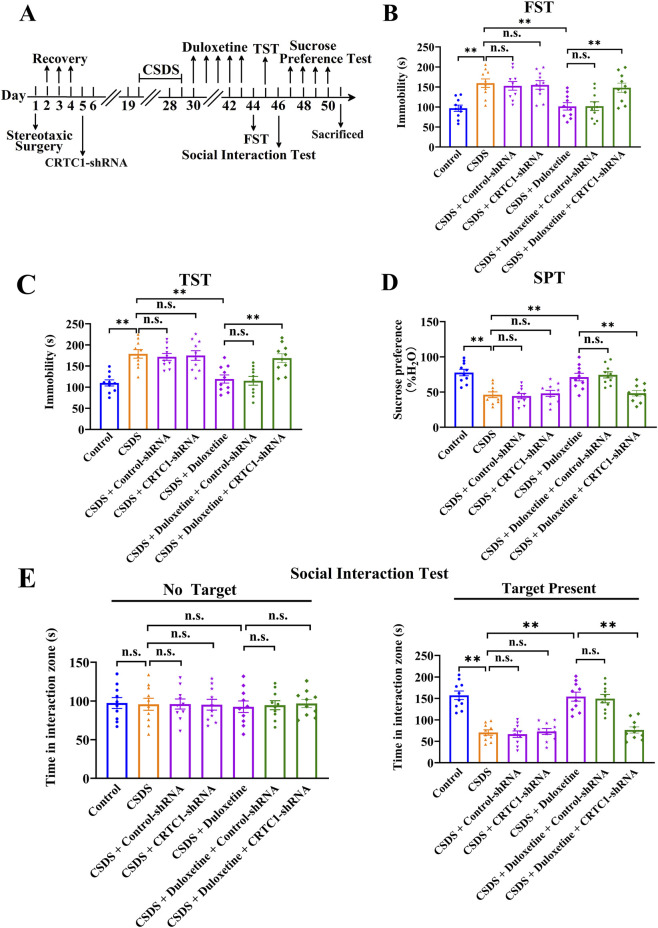
Hippocampal CRTC1 knockdown abolishes duloxetine’s effects in CSDS model. **(A)** Experimental timeline. **(B–E)** CRTC1 knockdown in the hippocampus significantly blocked the reversal effects of duloxetine on FST **(B)** immobility, TST **(C)** immobility, sucrose preference **(D)**, and social interaction **(E)** in mice exposed to CSDS (n = 10). Data are presented as mean ± S.E.M.; **p < 0.01, n. s., not significant. Two-way ANOVA and Bonferroni’s test were adopted together.

**FIGURE 7:**
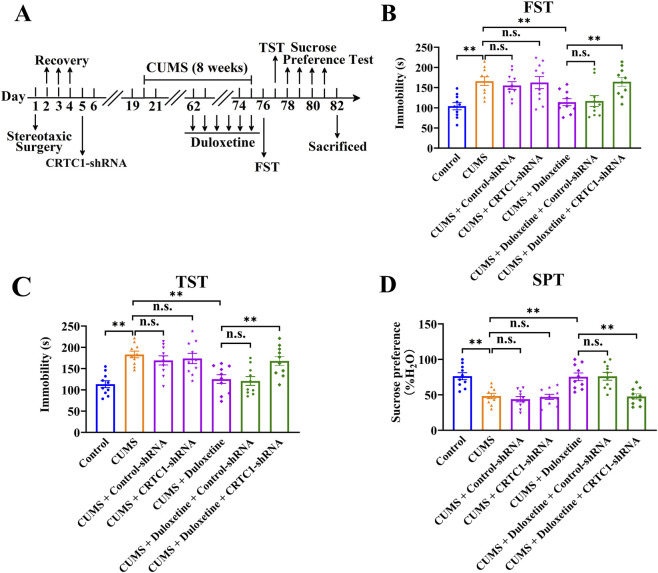
Hippocampal CRTC1 is required for duloxetine’s efficacy in CUMS model **(A)** Experimental timeline. **(B–D)** CRTC1 knockdown in the hippocampus notably prevented the reversal effects of duloxetine on FST **(B)** immobility, TST **(C)** immobility, and sucrose preference **(D)** in mice exposed to CUMS (n = 10). Data are presented as mean ± S.E.M.; **p < 0.01, n. s., not significant. Two-way ANOVA and Bonferroni’s test were adopted together.

**FIGURE 8:**
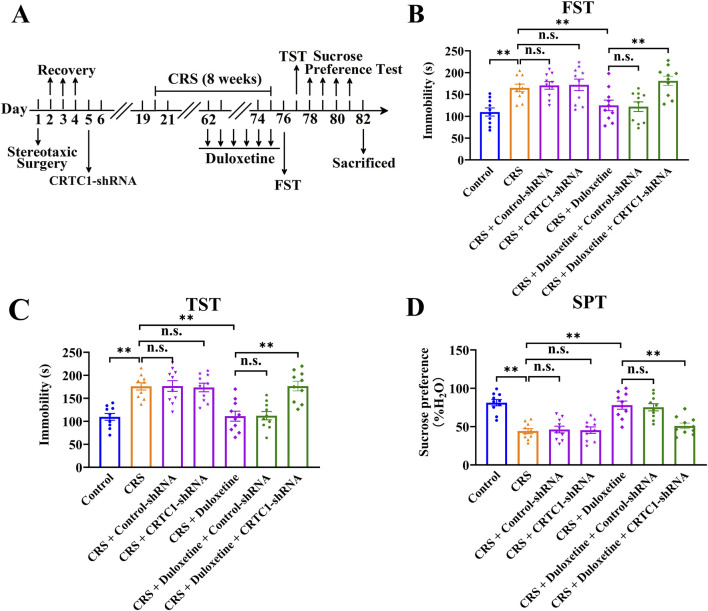
Hippocampal CRTC1 mediates duloxetine’s effects in CRS model. **(A)** Experimental timeline. **(B–D)** CRTC1 knockdown in the hippocampus evidently abrogated the reversal effects of duloxetine on FST **(B)** immobility, TST **(C)** immobility, and sucrose preference **(D)** in mice exposed to CRS (n = 10). Data are presented as mean ± S.E.M.; **p < 0.01, n. s., not significant. Two-way ANOVA and Bonferroni’s test were adopted together.

AAV-mediated CRTC1 knockdown reduced hippocampal CRTC1 protein expression by approximately 58% (n = 5, *p* < 0.01; Figures 5A,B). In CSDS-exposed mice, the antidepressant effects of duloxetine (20 mg/kg) were completely abolished by prior hippocampal CRTC1 knockdown. Mice receiving (CSDS exposure + duloxetine treatment + CRTC1-shRNA pre-infusion) showed no significant improvements in FST immobility [ANOVA: shRNA, F (1, 36) = 20.165, *p* < 0.01; Drug, F (1, 36) = 25.304, *p* < 0.01; Interaction, F (1, 36) = 17.866, *p* < 0.01], TST immobility [ANOVA: shRNA, F (1, 36) = 19.006, *p* < 0.01; Drug, F (1, 36) = 24.154, *p* < 0.01; Interaction, F (1, 36) = 15.261, *p* < 0.01], sucrose preference [ANOVA: shRNA, F (1, 36) = 16.539, *p* < 0.01; Drug, F (1, 36) = 19.135, *p* < 0.01; Interaction, F (1, 36) = 13.282, *p* < 0.01], or social interaction [ANOVA: shRNA, F (1, 36) = 26.489, *p* < 0.01; Drug, F (1, 36) = 32.062, *p* < 0.01; Interaction, F (1, 36) = 21.153, *p* < 0.01] compared to mice subjected to CSDS exposure solely (n = 10, *p* < 0.01; Figures 6A–E). Similarly, in CUMS-exposed mice, CRTC1 knockdown blocked duloxetine’s effects on FST immobility [ANOVA: shRNA, F (1, 36) = 23.095, *p* < 0.01; Drug, F (1, 36) = 27.237, *p* < 0.01; Interaction, F (1, 36) = 18.145, *p* < 0.01], TST immobility [ANOVA: shRNA, F (1, 36) = 23.967, *p* < 0.01; Drug, F (1, 36) = 26.658, *p* < 0.01; Interaction, F (1, 36) = 17.408, *p* < 0.01], and sucrose preference [ANOVA: shRNA, F (1, 36) = 19.132, *p* < 0.01; Drug, F (1, 36) = 23.084, *p* < 0.01; Interaction, F (1, 36) = 15.345, *p* < 0.01] (n = 10, *p* < 0.01; Figures 7A–D). In CRS-exposed mice, CRTC1 knockdown also prevented duloxetine’s behavioral benefits [ANOVA for FST: shRNA, F (1, 36) = 18.123, *p* < 0.01; Drug, F (1, 36) = 23.264, *p* < 0.01; Interaction, F (1, 36) = 14.151, *p* < 0.01. ANOVA for TST: shRNA, F (1, 36) = 20.801, *p* < 0.01; Drug, F (1, 36) = 25.471, *p* < 0.01; Interaction, F (1, 36) = 16.088, *p* < 0.01. ANOVA for SPT: shRNA, F (1, 36) = 15.932, *p* < 0.01; Drug, F (1, 36) = 21.685, *p* < 0.01; Interaction, F (1, 36) = 13.229, *p* < 0.01] (n = 10, *p* < 0.01; Figures 8A–D). Control-shRNA infusion did not affect duloxetine’s efficacy in any model (n = 10).

## 4 Discussion

The present study provides the first evidence that the serotonin-norepinephrine reuptake inhibitor duloxetine exerts its antidepressant effects through modulation of the hippocampal SIK2-CRTC1-CREB signaling pathway. Using three complementary chronic stress models, we demonstrate that duloxetine treatment normalizes chronic stress-induced upregulation of SIK2, restores CRTC1 nuclear translocation, and enhances CRTC1-CREB binding. Crucially, genetic ablation of hippocampal CRTC1 completely abolishes duloxetine’s behavioral efficacy, establishing this pathway as an essential mediator of its therapeutic action.

These findings extend our understanding of how SNRIs influence neuroplasticity beyond their immediate effects on monoamine reuptake. While duloxetine’s primary mechanism involves increasing synaptic availability of serotonin and norepinephrine, the delayed onset of clinical improvement—typically requiring several weeks—suggests that downstream adaptations in intracellular signaling cascades are necessary for therapeutic response (Brannan et al., 2005; Kirwin and Gören, 2005). The SIK2-CRTC1-CREB pathway represents one such adaptive mechanism, coupling monoaminergic input to long-term changes in gene expression that support neuronal function and resilience.

The convergence of multiple antidepressant classes on this pathway is noteworthy. Previous studies have implicated hippocampal SIK2-CRTC1 signaling in the actions of SSRIs (fluoxetine, paroxetine), SNRIs (venlafaxine), and other agents (mirtazapine) (Cai et al., 2024; Jiang et al., 2019). Our results add duloxetine to this list, suggesting that modulation of this pathway may represent a common downstream mechanism shared by diverse antidepressants. This convergence supports the view that the SIK2-CRTC1-CREB axis is a critical node in depression pathophysiology and a promising target for therapeutic intervention.

The precise mechanisms by which duloxetine regulates SIK2 expression and activity remain to be determined. Both serotonin and norepinephrine activate multiple G protein-coupled receptor subtypes coupled to distinct intracellular signaling pathways. 5-HT_4_ and 5-HT_7_ receptors stimulate adenylyl cyclase via G_s_, increasing cAMP and activating PKA, which can influence CREB-mediated transcription (Ahmed et al., 2022; Grueb et al., 2012; Tang et al., 2023; Yu et al., 2024; Zhang H. et al., 2020; Zixuan et al., 2025). Conversely, 5-HT_1A_ receptors coupled to G_i_ inhibit cAMP production (Albert and Vahid-Ansari, 2019; Ragini et al., 2023). Norepinephrine acting through β-adrenergic receptors also activates the cAMP-PKA cascade (Liu et al., 2019; Sun et al., 2018). These pathways may converge on transcriptional regulators of SIK2 expression or on post-translational modifications affecting SIK2 stability (Henriksson et al., 2012; Paulo et al., 2018; Ricarte et al., 2018; Shi et al., 2025). The observation that duloxetine normalized both SIK2 protein and mRNA levels suggests transcriptional regulation, possibly through feedback mechanisms involving CREB itself.

An intriguing aspect of our findings is the pathology-dependent nature of duloxetine’s effects. Duloxetine altered SIK2 and CRTC1 expression in stressed mice but had no effect in non-stressed controls, indicating that it acts to normalize dysregulated signaling rather than broadly perturbing basal function. This selectivity may contribute to duloxetine’s favorable tolerability profile in clinical use (Rodrigues-Amorim et al., 2020). The mechanisms underlying this stress-dependent action warrant further investigation but may involve stress-induced changes in receptor expression, signaling efficiency, or chromatin accessibility that render the pathway susceptible to modulation.

Our knockdown experiments provide strong causal evidence for CRTC1 requirement. The complete loss of duloxetine’s behavioral effects following hippocampal CRTC1 depletion indicates that this molecule is not merely correlated with antidepressant response but is functionally necessary. Given CRTC1’s role as a CREB coactivator regulating BDNF and other plasticity-related genes (Yan et al., 2021), these findings align with the neurotrophic hypothesis of depression and suggest that duloxetine’s therapeutic effects ultimately depend on enhancing CREB-dependent transcription.

Several limitations should be acknowledged. First, the exclusive use of male mice represents a significant limitation of this study. Major depressive disorder exhibits a pronounced sex bias, with women approximately twice as likely as men to be diagnosed (Bangasser and Cuarenta, 2021). Preclinical evidence has demonstrated sex differences in stress responses and antidepressant efficacy (including duloxetine) (Xu et al., 2017). Importantly, the SIK2-CRTC1-CREB pathway itself may be subject to sex-specific regulation. Estrogen signaling can modulate CREB activity and CRTC family coactivator expression (Jiang et al., 2021; Samarajeewa et al., 2013). Whether duloxetine’s engagement of the SIK2-CRTC1 pathway differs between males and females remains entirely unknown. While the present proof-of-principle study in males establishes that this pathway is sufficient for duloxetine’s antidepressant effects, future investigations using female mice will be essential to determine whether this mechanism is also necessary and sufficient in females. Such studies are currently underway in our laboratory. Second, while we demonstrate CRTC1 requirement, the upstream receptors and signaling molecules linking duloxetine to SIK2 regulation remain to be identified. Third, our focus on the hippocampus does not exclude involvement of other limbic regions such as prefrontal cortex or amygdala. Fourth, although we show restoration of CRTC1-CREB binding, the full complement of CREB target genes affected by duloxetine treatment requires further characterization. Fifth, direct measurement of duloxetine brain exposure was not performed in this study. While i. p. administration is a widely accepted route in preclinical antidepressant research, and the duloxetine-induced behavioral effects along with significant molecular alterations in hippocampal tissue indirectly support effective central exposure, we acknowledge that brain concentration data would provide more direct pharmacokinetic-pharmacodynamic correlation. Future studies employing liquid chromatography-tandem mass spectrometry (LC-MS/MS) to measure hippocampal duloxetine concentrations will be valuable to establish the relationship between brain exposure and the SIK2-CRTC1 pathway modulation reported here.

In conclusion, this study identifies the hippocampal SIK2-CRTC1-CREB pathway as a critical mediator of duloxetine’s antidepressant effects. By demonstrating that duloxetine normalizes chronic stress-induced dysregulation of this signaling cascade and that CRTC1 is essential for its behavioral efficacy, we provide new mechanistic insight into how SNRIs promote neuroplasticity and resilience. These findings reinforce the therapeutic potential of targeting this pathway for depression treatment and contribute to the growing understanding of antidepressants as modulators of intracellular signaling beyond their classical effects on monoamine neurotransmission.

## Data Availability

The raw data supporting the conclusions of this article will be made available by the authors, without undue reservation.
