# Supplementary figures and images for: Duloxetine ameliorates chronic stress-induced depressive behaviors by normalizing hippocampal SIK2-CRTC1 signaling
Source: Front Pharmacol. 2026 Jul 2;17:1836082. doi: 10.3389/fphar.2026.1836082 (PMC13373589; doi:10.3389/fphar.2026.1836082)

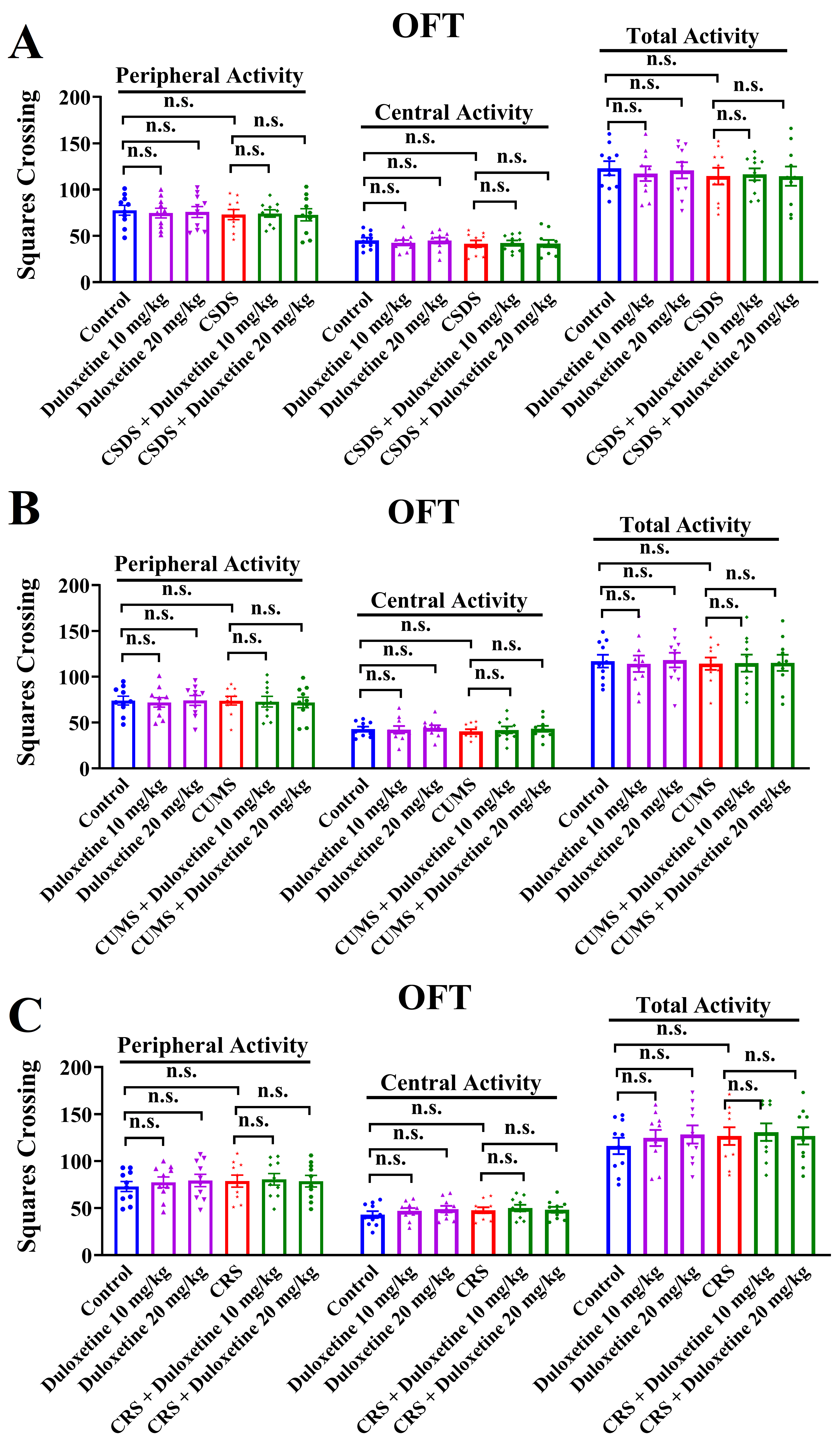

Supplement: Supplementary file 1 [file Image1.jpeg]
