# Supplementary material for: Duloxetine ameliorates chronic stress-induced depressive behaviors by normalizing hippocampal SIK2-CRTC1 signaling
Source: Front Pharmacol. 2026 Jul 2;17:1836082. doi: 10.3389/fphar.2026.1836082 (PMC13373589; doi:10.3389/fphar.2026.1836082)
Supplement: Supplementary file 2 [file DataSheet1.docx]

**Supplementary Information**

**Materials and Methods**

*Chronic Unpredictable Mild Stress (CUMS)*

The CUMS paradigm was established based on reported protocols (Cai et al., 2024; Huang et al., 2023; Jiang et al., 2019; Liu et al., 2020). Individually housed C57BL/6J mice underwent an 8-week regimen featuring two distinct, mild stressors applied daily in a random sequence. These stressors comprised: tilting cages at a 45° angle (12 h), using moist bedding (12 h), immobilizing in a restraint tube (1 h), exposure to constant light (12 h), withdrawal of food and water (23 h), exposure to white noise at 85 dB (4 h), exposure to cold environment at 4°C (1 h), rotating the cages (30 min), and reversing the light/dark cycle overnight. Control animals were maintained in group housing and received daily handling. Pharmacological agents were given once daily (between 8:00-9:00 AM) during the concluding two weeks of the stress protocol, and behavioral tests were conducted 24 hours after the final injection.

*Chronic Restraint Stress (CRS)*

For CRS experiments, we followed previously described methods (Tian et al., 2024; Zhao et al., 2025). Mice assigned to the stress group were individually restrained for 3 hours daily (9:00 a.m. to 12:00 p.m.) over an 8-week period, with drug administration occurring once daily (between 8:00-9:00 AM) during the final two weeks. Behavioral tests were conducted 24 hours after the final injection. Restraint was implemented using 50 ml conical plastic tubes equipped with ventilation openings. Control mice received only routine handling.

*Forced Swim Test (FST)*

As per established procedures (Cai et al., 2024; Huang et al., 2023; Jiang et al., 2019; Liu et al., 2020), animals were placed into transparent plexiglas cylinders (height: 25 cm, diameter: 10 cm) containing water at a depth of 15 cm (temperature maintained at 24 ± 1°C). The 6-minute trial was videotaped. An experimenter unaware of the experimental groups manually quantified the duration of immobility, characterized by passive floating with only slight movements required for keeping the nose above the water surface, during the last 4 minutes.

*Tail Suspension Test (TST)*

Following published guidelines (Cai et al., 2024; Huang et al., 2023; Jiang et al., 2019; Liu et al., 2020), mice were hung by their tails with adhesive tape applied approximately 2 cm from the tail tip to a bar positioned 50 cm above the surface. The 6-minute trial was captured on video, and a blinded observer measured the total immobility time, defined as the absence of any limb or bodily motion.

*Sucrose Preference Test (SPT)*

Anhedonia was evaluated via a two-bottle choice test, consistent with prior reports (Cai et al., 2024; Huang et al., 2023; Jiang et al., 2019; Liu et al., 2020). Following a 48-hour habituation period to 1% sucrose solution, animals experienced an 18-hour period without food and water. Subsequently, they were given access to two pre-weighed bottles for 6 hours—one containing 1% sucrose solution and the other containing tap water. To mitigate side bias, the bottle positions were swapped at the midpoint (3 h). Sucrose preference was derived from the formula: [Sucrose intake (g) / (Sucrose + Water intake (g))] × 100%.

*Social Interaction Test*

Social avoidance was evaluated using a two-phase procedure within an open-field arena (42 × 42 cm), as detailed in earlier work (Cai et al., 2024; Jiang et al., 2019; Liu et al., 2020). During the initial 5-minute "target absent" phase, the subject mouse was allowed to freely explore the arena containing an empty wire mesh cage. In the subsequent 5-minute "target present" phase, an unfamiliar CD1 mouse was placed inside the cage. The duration spent in the interaction zone (a 14 × 26 cm region adjacent to the cage) for each phase was measured automatically with EthoVision XT tracking software (Noldus).

*Open field test (OFT)*

Enhanced locomotor activity in rodents may contribute to reduction of immobility duration in the FST and TST, leading to false-positive conclusion (Bourin et al., 2001). To exclude this possibility, the OFT was used. In brief, mice were individually introduced into an open field apparatus (100 × 100 × 45 cm; 25 squares) for a 5-min period. The apparatus was illuminated with a red bulb (50 W) on the ceiling. The number of peripheral and central squares each animal crossed during the whole period was individually recorded an observer unaware of animal grouping under a dim light environment.

*Western Blotting*

Consistent with established methods (Cai et al., 2024; Huang et al., 2023; Jiang et al., 2019; Liu et al., 2020), hippocampal samples were quickly excised on ice and homogenized in RIPA buffer (Beyotime, Shanghai, China) containing protease and phosphatase inhibitor cocktails (Roche, Basel, Switzerland). Cytoplasmic and nuclear extracts were separated utilizing a NE-PER kit (Thermo Fisher, Waltham, USA). Protein content was measured with the BCA assay. Equivalent protein quantities (30 µg) underwent separation via 10/12% SDS-PAGE and were subsequently transferred onto PVDF membranes (Millipore, Billerica, USA). After blocking with 5% nonfat milk, membranes were incubated overnight at 4°C with primary antibodies targeting: SIK2 (1:1000; Cell Signaling, Danvers, USA), CRTC1 (1:1000; Cell Signaling), phospho-CRTC1 (Ser151) (1:500; Millipore), CREB (1:1000; Cell Signaling), histone H3 (1:1000; Cell Signaling), and β-actin (1:5000; Cell Signaling). Following exposure to HRP-linked secondary antibodies, protein bands were visualized using an ECL substrate (Bio-Rad) and quantified with Image Lab software (Bio-Rad). Signals were normalized to appropriate loading controls (β-actin for whole-cell lysates/cytoplasmic fractions, histone H3 for nuclear fractions).

*Quantitative Real-time Reverse Transcription PCR (qRT-PCR)*

As per standard protocols (Cai et al., 2024; Huang et al., 2023; Jiang et al., 2019; Liu et al., 2020), hippocampal tissues from each C57BL/6J mouse were harvested in chilled antifreeze solution post-euthanasia. Total RNA was isolated employing TRIzol™ reagent (Thermo Fisher), and its concentration was assessed with a Nanodrop 2000 Spectrophotometer (Thermo Fisher). Reverse transcription into cDNA was accomplished using PrimeScript™ RT Master Mix (Takara, Tokyo, Japan). Quantitative PCR was subsequently performed on a Step-One-Plus™ Real-Time PCR System (Thermo Fisher) utilizing SYBR® fast qPCR master mix (Takara). Glyceraldehyde-3-phosphate dehydrogenase (GAPDH) served as the internal reference, with each sample run in triplicate. PCR primers (listed below) were synthesized by Sangon Biotech (Shanghai, China). The cycle threshold (CT) was determined, and relative expression levels of SIK2 and CRTC1 mRNA were calculated using the △△CT method, with normalization to GAPDH.

| Primers List | Forward | Reverse |
| --- | --- | --- |
| SIK2 mRNA | 5’-TCAAGGAGCACAAGTGGAT  G-3’ | 5’-TGCATCAGTCGAAGAACC  TG -3’ |
| CRTC1 mRNA | 5’-TCTCCGGTCTCCAACCAAG  GC-3’ | 5’-CTGGCTGTCATCTGCTGCT  CATAG-3’ |
| GAPDH mRNA | 5’-ACATTGTTGCCATCAACGA  C-3’ | 5’-ACGCCAGTAGACTCCACG  AC-3’ |

*Co-immunoprecipitation (Co-IP)*

To examine the interaction between CRTC1 and CREB, we followed previously reported procedures (Cai et al., 2024; Huang et al., 2023; Jiang et al., 2019; Liu et al., 2020). Hippocampal lysates containing 500 µg protein were precleared with Protein A/G agarose beads (Santa Cruz Biotech, Santa Cruz, USA) for 1 hour at 4°C. The clarified supernatants were then incubated overnight at 4°C with 2 µg of either anti-CRTC1 or anti-CREB antibody (Cell Signaling). Immune complexes were precipitated by adding Protein A/G agarose beads for 2 hours at 4°C, washed four times using lysis buffer, and eluted with 2× Laemmli sample buffer. Western blotting with anti-CRTC1 or anti-CREB antibodies was used to detect co-precipitated proteins. Input controls (10% of the lysate used for immunoprecipitation) were included alongside.

*Stereotaxic Surgery and Viral-mediated Gene Knockdown*

Mice were anesthetized with sodium pentobarbital (50 mg/kg, i.p.) and positioned in a stereotaxic frame (RWD Life Science, Shenzhen, China). Adeno-associated virus serotype 9 (AAV9) vectors carrying short hairpin RNA (shRNA) under the U6 promoter along with enhanced green fluorescent protein (EGFP) under a separate CAG promoter were employed. AAV-CRTC1-shRNA and a non-targeting AAV-Control-shRNA were sourced from Genechem Co., Ltd. (Shanghai, China). Viral suspensions (titer: 2 × 10¹² viral genomes/ml) were bilaterally administered into the dorsal hippocampus (stereotaxic coordinates relative to bregma: AP - 2.0 mm, ML ± 1.5 mm, DV - 1.8 mm (Cai et al., 2024; Huang et al., 2023; Jiang et al., 2019; Liu et al., 2020)) at an infusion rate of 0.5 µl/min (1.5 µl per hemisphere) using a double-guide cannula (Plastics One, USA) linked to a 10 µl Hamilton syringe. The injector was kept in position for an additional 5 minutes after infusion to facilitate diffusion. A 14-day recovery period was allowed for optimal viral expression prior to subsequent experimentation. The sequences for control-shRNA and CRTC1-shRNA were 5’-TTCTCCGAACGTGTCA

CGT-3’ and 5’-GCAGTTCAACATGATGGAGAA-3’, respectively.

References

Bourin, M., Fiocco, A.J., Clenet, F., 2001. How valuable are animal models in defining antidepressant activity? Hum Psychopharmacol 16, 9-21.

Cai, X.M., Sun, X.Y., Li, R., Wang, P.J., Qiu, J.C., Ge, Y.X., Yang, L., 2024. The hippocampal salt-inducible kinase 2-CREB-regulated transcription co-activator 1 system mediates the antidepressant actions of paroxetine in mice. Behav Brain Res 465, 114972.

Huang, J., Fan, H., Chen, Y.M., Wang, C.N., Guan, W., Li, W.Y., Shi, T.S., Chen, W.J., Zhu, B.L., Liu, J.F., Jiang, B., 2023. The salt-inducible kinases inhibitor HG-9-91-01 exhibits antidepressant-like actions in mice exposed to chronic unpredictable mild stress. Neuropharmacology 227, 109437.

Jiang, B., Wang, H., Wang, J.L., Wang, Y.J., Zhu, Q., Wang, C.N., Song, L., Gao, T.T., Wang, Y., Meng, G.L., Wu, F., Ling, Y., Zhang, W., Li, J.X., 2019. Hippocampal Salt-Inducible Kinase 2 Plays a Role in Depression via the CREB-Regulated Transcription Coactivator 1-cAMP Response Element Binding-Brain-Derived Neurotrophic Factor Pathway. Biol Psychiatry 85, 650-666.

Liu, Y., Tang, W., Ji, C., Gu, J., Chen, Y., Huang, J., Zhao, X., Sun, Y., Wang, C., Guan, W., Liu, J., Jiang, B., 2020. The Selective SIK2 Inhibitor ARN-3236 Produces Strong Antidepressant-Like Efficacy in Mice via the Hippocampal CRTC1-CREB-BDNF Pathway. Front Pharmacol 11, 624429.

Tian, X., Wang, G., Teng, F., Xue, X., Pan, J., Mao, Q., Guo, D., Song, X., Ma, K., 2024. Zhi Zi Chi decoction (Gardeniae fructus and semen Sojae Praeparatum) attenuates anxious depression via modulating microbiota-gut-brain axis in corticosterone combined with chronic restraint stress-induced mice. CNS Neurosci Ther 30, e14519.

Zhao, F., Piao, J., Song, J., Geng, Z., Chen, H., Cheng, Z., Cui, R., Li, B., 2025. Traditional Chinese herbal formula, Fuzi-Lizhong pill, produces antidepressant-like effects in chronic restraint stress mice through systemic pharmacology. J Ethnopharmacol 338, 119011.

**Figure Legends**

Figure S1. CSDS exposure (A), CUMS exposure (B), CRS exposure (C), and i.p. injection of duloxetine all did not affect the locomotor activity of mice, as revealed by the OFT. No significant differences were found among all groups in the number of squares that a mouse crossed in the central or peripheral area (n = 10). All results are presented as mean ± S.E.M; n.s., no significance.
